# Keratin 8/18 Regulate the Akt Signaling Pathway

**DOI:** 10.3390/ijms22179227

**Published:** 2021-08-26

**Authors:** Younglan Lim, Sujin Kim, Han-Na Yoon, Nam-On Ku

**Affiliations:** 1 Interdisciplinary Program of Integrated OMICS for Biomedical Sciences, Yonsei University, Seoul 03722, Korea; kr_ran@hanmail.net (Y.L.); kimsj8811@gmail.com (S.K.); dbsgkssksla@hanmail.net (H.-N.Y.); 2 Department of Bio-Convergence ISED, Underwood International College, Yonsei University, Seoul 03722, Korea

**Keywords:** Akt, keratin 8, keratin 18, mouse model, liver

## Abstract

Keratin 8 and keratin 18 (K8/K18) are intermediate filament proteins that form the obligate heteropolymers in hepatocytes and protect the liver against toxins. The mechanisms of protection include the regulation of signaling pathway associated with cell survival. Previous studies show K8/K18 binding with Akt, which is a well-known protein kinase involved in the cell survival signaling pathway. However, the role of K8/K18 in the Akt signaling pathway is unclear. In this study, we found that K8/K18-Akt binding is downregulated by K8/K18 phosphorylation, specifically phosphorylation of K18 ser7/34/53 residues, whereas the binding is upregulated by K8 gly-62-cys mutation. K8/K18 expression in cultured cell system tends to enhance the stability of the Akt protein. A comparison of the Akt signaling pathway in a mouse system with liver damage shows that the pathway is downregulated in K18-null mice compared with nontransgenic mice. K18-null mice with Fas-induced liver damage show enhanced apoptosis combined with the downregulation of the Akt signaling pathway, i.e., lower phosphorylation levels of GSK3β and NFκB, which are the downstream signaling factors in the Akt signaling pathway, in K18-null mice compared with the control mice. Our study indicates that K8/K18 expression protects mice from liver damage by participating in enhancing the Akt signaling pathway.

## 1. Introduction

Keratins belong to the intermediate filament proteins and are divided into two groups, type I (K9-K28, K31-K40) and type II (K1-K8, K71-K86) keratins, which form obligate heterodimers, and thus, the single expressed keratin is unstable and easily degraded [1,2,3]. The heterodimers consist of different subtypes of keratins in a tissue-specific manner [2]. In the liver, K8 and K18 are mainly expressed, while in the colon, K7 and K8 are expressed together with K18, K19, and K20 [2]. The roles of K8 and K18 are studied in the knock-out mouse model. K8 knock-out caused high embryonic lethality and spontaneous colitis in mice and some of the K8-null mice showed damages in the liver and spleen even without any other external stimulation [4,5]. Under liver-specific stimulation, K8 null mice showed more severe damage compared to K8-expressing mice [6]. In the case of K18-null mice, they were not different from the control mice under the basal conditions, but some of the old K18 knock-out mice had abnormal aggregates in the liver [7]. K18-null mice seemed healthy without external stresses, but the mice were highly susceptible to external stimulation, such as Fas-induced liver injury, as compared to the nontransgenic mice [6]. Although K8/K18-mediated liver protection was described in the studies using K8- or K18-null mice, the detailed mechanisms relating to the protection need to be elucidated.

Akt was originally found from v-Akt oncogene in the Akt8 transforming retrovirus [8], and the cellular homolog of v-Akt was cloned [9]. It was named protein kinase B (PKB), as it had a similarity to PKA and PKC [10]. As a serine/threonine kinase, Akt is activated by external signals through membrane receptor activation [11]. When membrane receptors, such as receptor tyrosine kinase and G protein-coupled receptors, are stimulated, phosphoinositide 3-kinase (PI3K) is also activated. The activated PI3K phosphorylates PIP_2_ to PIP_3_, and then PDK and Akt are activated in order. Finally, the activated Akt phosphorylates its substrates, such as GSK3β, BAD, and mTORC. There are over 100 reported substrates of Akt [12].

Akt regulates various biological responses, such as cell growth, motility, proliferation, and metabolism [12]. Among the diverse functions of Akt, Akt is involved in cell survival. A previous study demonstrated that when Akt1 was knocked out, mice showed retarded growth, and their life span was shorter compared to WT mice [13]. In addition, the mouse embryo fibroblasts from Akt1 null mice were more vulnerable to apoptosis-inducing stresses [13]. This study showed that Akt is involved in cell proliferation as well as cell survival. Furthermore, Akt protects cells by blocking pro-apoptotic proteins or enhancing survival pathways. For instance, BAD normally blocks Bcl-2 and Bcl-x_L_ by interacting with it, but BAD loses the ability to bind Bcl-2 and Bcl-x_L_ after being phosphorylated by Akt [14]. Eventually, free Bcl-2 and Bcl-x_L_ act as anti-apoptotic factors and protect cells from apoptosis, indicating which Akt inhibits apoptosis by phosphorylating its substrates. Alternatively, Akt activates NF-kB by phosphorylating IkB kinase (IKK) [15]. By the crosstalk between the PI3K/Akt pathway and NF-kB pathway, Akt functions as a survival effector.

It has been reported that Akt could interact with intermediate filaments, such as vimentin, lamin A, and keratin 10 (K10) [16,17,18,19]. In the case of vimentin, Akt–vimentin binding resulted in the phosphorylation of vimentin S39 that led to increasing cell migration and tumor invasion [16]. Moreover, vimentin is a regulator of tumorigenesis. Its expression was upregulated by the activation of the PI3K/Akt signaling pathway in oral carcinoma [20], and it was also involved in the inhibition of autophagy by the Akt-mediated mechanism in the progression of lung cancer [21]. Lamin A is also known as an Akt substrate [17]. Lamin A S404 is phosphorylated by Akt, and when the phosphorylation was blocked by the point mutation, nuclear abnormalities appeared [17]. Not only the point mutation of S404, but also the point mutation of R401 made nuclei vulnerable, since R401 was the amino acid in the Akt consensus sequence (RXXpS/pT) [17]. It was reported that lamin A could aggravate prostate cancer by regulating the PI3K protein level and Akt activity [22].

Some keratins also interact with Akt. Unlike vimentin and lamin A, K10 was not known as a substrate of Akt. However, it was reported that K10 interacted with Akt through its *N*-terminal domain [18] and the K10 inhibited cell proliferation and tumorigenesis by downregulating Akt [18,19]. In the case of K17, it was unrevealed whether K17 interacted with Akt or not. Nonetheless, K17 has an effect on Akt activity [23,24]. Decreased expression of K17 resulted in decreased activity of Akt and mTOR and eventually a lower rate of proliferation compared to the normally expressed conditions [23,24]. Some intermediate filaments are substrates of Akt, and the others are not revealed as substrates. However, the intermediate filaments somehow seem to be involved in Akt regulation. To understand the diverse regulation of the Akt signaling pathway, it is necessary to further investigate intermediate filaments, including keratins. Considering the wide range of keratins, discovering the effects of keratins on Akt may contribute to revealing the unknown regulation of the Akt signaling pathway.

A previous study reveals that Akt can interact with K8/K18, and their binding is not affected by the mutation of Akt T308 or glycosylation of K8/K18 [25]. However, the binding conditions or roles of their interaction have not been studied to date. Herein, we found that the interaction of Akt with K8/K18 is regulated by phosphorylation—specifically, K18 phosphorylation—and K8/K18 expression increased Akt activity followed by Akt substrate activity, such as GSK3β and NFκB.

## 2. Results

### 2.1. K18 Phosphorylation Inhibits K8/K18 Interaction with Akt Kinase

**Figure 1:**
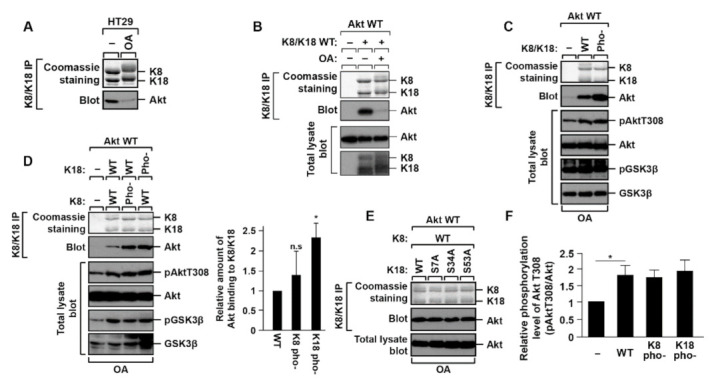
K8/K18–Akt interaction is downregulated by K18 S7/34/53 phosphorylation. (**A**) HT29 cells were treated with okadaic acid (OA, 1 µM) for 2 h, and then Akt interaction was tested by immunoprecipitation against K8/K18 followed by immunoblotting with Akt antibody. (**B**) BHK-21 cells were transfected with Akt WT and K8/K18 WT, and treated with OA (0.5 µM) for 2 h. K8/K18 immunoprecipitates and total lysates were prepared and immunoblotted with the indicated antibodies. (**C**) K8/K18 WT or phosphorylation-deficient mutants (pho-) were transfected into BHK-21 cells together with Akt WT, and then OA was treated 2 h before harvesting cells. K8/K18–Akt interaction was tested by coimmunoprecipitation assay, and cell lysates were immunoblotted with phosphorylated Akt and phosphorylated GSK3 antibodies. K8/K18 pho- indicates K8 S21/22/24/37/43/74/432/451A and K18 S7/34/53A. (**D**) BHK-21 cells were transfected with the indicated keratin constructs together with Akt WT. Immunoprecipitation was performed with the cells after a 2-h treatment of OA. Each keratin construct is represented as follows: K8 pho-, K8 S21/22/24/37/43/74/432/451A; K18 pho-, K18 S7/34/53A. The graph represents the means ± S.E. of three independent experiments. * indicates *p* < 0.05. ‘n.s.’ indicates ‘not significant’. (**E**) Akt WT and K8 WT were transfected into BHK-21 cells with the K18 single-site phosphorylation-deficient mutant (K18 S7A, K18 S34A, or K18 S53A). The cells were treated with OA for 2 h, and the Akt interaction with K8/K18 was tested by the immunoprecipitation assay. (**F**) The transfected BHK-21 cells were prepared as described in panel D. Akt activity was determined by immunoblotting cell lysates with antibody against phosphorylated Akt T308. The data were obtained from three independent experiments, and the mean values ± S.E. are shown in the graph. * indicates *p* < 0.05.

A previous study revealed that Akt interacted with the K8/K18 complex, and K18 glycosylation or Akt T308 phosphorylation has no effect on Akt-binding to K8/K18 [25]. However, the binding conditions or roles of the interaction were not studied. To determine the binding conditions, the HT29 cell line was used since HT29 cells express K8/K18 endogenously. When the phosphorylation level was increased in the cells after treatment of the phosphatase inhibitor, such as okadaic acid (OA), Akt was separated from K8/K18 (Figure 1A). To reconfirm the detachment of Akt from K8/K18 under the OA treatment conditions, the transient transfection system was used with BHK-21 cells, which did not express endogenous K8 and K18. The cells were transfected with K8/K18 WT and Akt WT followed by OA treatment. The interaction between Akt and keratins was examined by an immunoprecipitation assay. The Akt binding to K8/K18 was decreased under OA treatment conditions (Figure 1B). These experiments with two different cell lines show that the increased level of cellular phosphorylation could affect the interaction between Akt and K8/K18. In addition, we examined whether other post-translational modifications, such as acetylation and methylation, affect the interaction between Akt and K8/K18. HT29 cells were treated with histone deacetylase inhibitors (MS-275, TSA, and nicotinamide) or methylation enhancers (hemin and CORM) to enhance the level of acetylation or methylation, respectively. The results show that these post-translational modifications have no effect on the K8/K18–Akt interaction (Supplementary Figure S1).

A previous study demonstrated that Akt T308 phosphorylation could not affect the K8/K18–Akt interaction [25]. Here, we tested whether K8/K18 phosphorylation has an effect on their binding. We used keratin mutants whose serine (S) residues that were supposed to be phosphorylated were substituted to alanine (A): phosphorylation-deficient K8 mutant, K8 S21/22/24/37/43/74/432/451A (K8 pho-), and phosphorylation-deficient K18 mutant, K18 S7/34/53A (K18 pho-). BHK-21 cells were transfected with the phosphorylation-deficient mutants of K8 and/or K18 together with Akt (Figure 1C,D). The phosphorylation-deficient mutations of K8/K18 caused an increase in the interaction of the K8/K18 with Akt (Figure 1C). To define whether K8 or K18 phosphorylation affects the interaction, a K8 or K18 phosphorylation-deficient mutant was transfected with K18 WT or K8 WT, respectively, and then Akt binding was examined by co-immunoprecipitation. The K18 S7/34/53A mutant (K18 pho-) showed a statistically significant increased interaction with Akt compared with the control or K8 phosphorylation-deficient mutant (Figure 1D). However, K18 single phosphorylation site mutations (K18 S7A, S34A, or S53A) did not show a significant change in their binding to Akt (Figure 1E). Additionally, we then tested whether the increased K8/K18–Akt interaction (in the case of K18 pho-) regulates Akt T308 phosphorylation that leads to Akt activation. In our tested conditions, we could not detect any significant correlation between K8/K18–Akt interaction and Akt activation/inactivation (Figure 1F). However, co-immunoprecipitation experiments clearly showed that the interaction between Akt and K8/K18 was altered by keratin phosphorylation, specifically, K18 phosphorylation.

### 2.2. K8 and K18 as Potential Substrates of Akt

**Figure 2:**
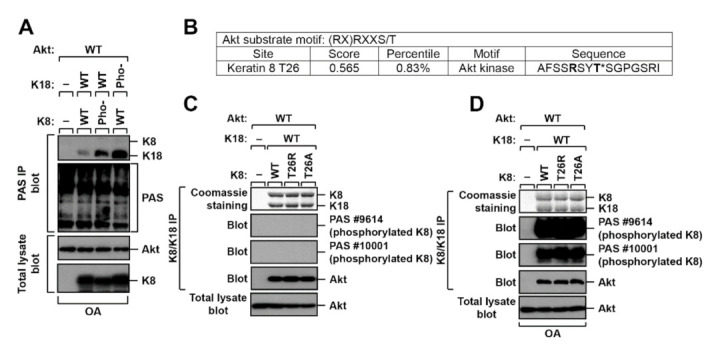
K8/K18 are potential substrates of Akt. (**A**) A K8 or K18 phosphorylation-deficient mutant was transfected into BHK-21 cells, and the cells were treated with OA for 2 h. The transfected cells were immunoprecipitated against phosphorylated Akt substrate (PAS) antibodies followed by immunoblotting with K8/K18. (**B**) The Akt substrate motif was analyzed in K8 protein sequences by Scansite (scansite4.mit.edu). K8 T26 was shown as a potential Akt substrate. (**C**,**D**) BHK-21 cells were transfected with K8 WT, T26R, or T26A together with K18 WT and Akt WT and then cultured under basal (**C**) or OA treatment (**D**) conditions. The cells were immunoprecipitated against K8/K18, and then the immunoprecipitates were immunoblotted with PAS antibodies. Each PAS antibody detects the following consensus sequence: PAS #9614, RXXpS/pT; PAS #10001, RXRXXpS/pT.

Since Akt is a well-known serine/threonine kinase, we suspected K8/K18 as substrates of Akt. The phosphorylation-deficient mutant of K8 or K18 was transfected into BHK-21 cells followed by OA treatment, and the cells were immunoprecipitated with phosphorylated Akt substrate (PAS) antibody. The PAS antibody detects phosphorylated serine (S) or threonine (T) residues in the Akt phosphorylation consensus sequences (RXRXXpS/pT or RXXpS/pT) [26]. Immunoblotting with PAS antibody showed various Akt substrates that are phosphorylated in the consensus sequences (Figure 2A). Additionally, the PAS immunoprecipitates were then blotted with an antibody of K8/K18 to determine if K8/K18 are substrates of Akt. It seems that K18, specifically, phosphorylation-deficient K18 mutant (K18 pho-), is a better substrate of Akt compared with K8 under the testing conditions (Figure 2A).

Since the keratin phosphorylation-deficient mutants had increased affinity with Akt (Figure 1D), it could be suspected that some residues, unknown phosphorylation sites, in K8/K18 might be the targets of Akt, and thus, the association of Akt and K8/K18 increased Akt-mediated phosphorylation. Therefore, we analyzed the K8/K18 protein sequences with Scansite (scansite4.mit.edu) to determine if K8 or K18 could be a substrate of Akt. Unexpectedly, the sequence analysis showed that K8 T26 residue, but not a residue in K18, was a potential Akt substrate (Figure 2B). Thus, we generated the K8 T26A mutant to block the possible phosphorylation, and K8 T26R, which was identified in patients with liver disease [27]. BHK-21 cells were transfected with K8 WT, T26A, or T26R, together with Akt WT and K18 WT. K8/K18 immunoprecipitates were immunoblotted with the PAS antibody to verify if K8 T26 residue was the substrate of Akt. The result showed that the PAS antibody did not detect K8 or K18 under basal conditions (Figure 2C), whereas the antibody detected K8, independent of the mutation of the T26 residue, under the OA treatment conditions (Figure 2D). Although the Akt-mediated phosphorylation sites on K8 or K18 could not be detected under the testing conditions, there was a possibility that K8/K18 could be the substrates of Akt since PAS antibodies detected K8 (Figure 2D) and K18 (Figure 2A).

### 2.3. K8 G62C, R302C, and G434S Mutations, Found in Patients with Liver Diseases, Cause an Increase in the K8/K18–Akt Interaction

**Figure 3:**
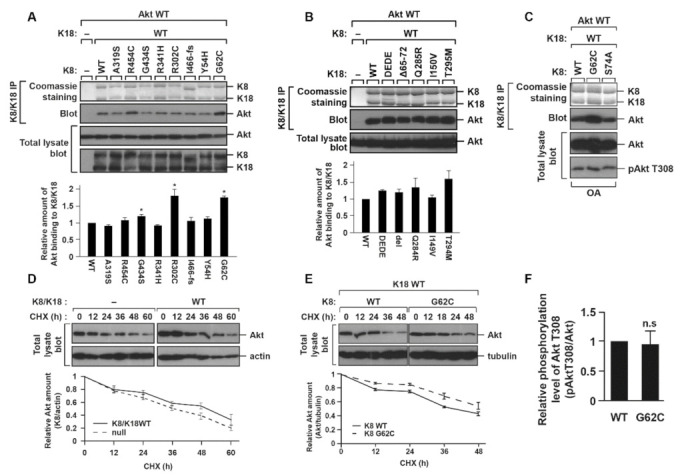
K8 G62C mutation enhanced K8/K18 binding with Akt. (**A**) BHK-21 cells were transfected with Akt, K18 WT, and one of the indicated K8 mutants identified in patients with liver diseases. The cell lysates were immunoprecipitated against K8/K18, and the K8/K18 immunoprecipitates were immunoblotted with antibody against Akt to verify the K8/K18–Akt interaction. The graph represents the means ± S.E. from three independent experiments. * indicates *p* < 0.05. (**B**) BHK-21 cells were transfected with Akt, K8 WT, and one of the indicated K18 mutants found in patients with liver diseases or the caspase-resistant mutant. The K18 D238/396E (DEDE) mutant is resistant to caspase-induced K18 cleavage. The K8/K18 immunoprecipitates were prepared and immunoblotted as described in panel A. The graph represents the means ± S.E. of three independent experiments. (**C**) BHK-21 cells were transfected with Akt WT, K18 WT, and one of K8 constructs (K8 WT, K8 G62C, or K8 S74 phospho-deficient mutant (K8 S74A). The cells were treated with OA, and then K8/K18 immunoprecipitates and total lysates were immunoblotted with antibody to the indicated epitopes. (**D**) BHK-21 cells or transfected BHK-21 cells with K8/K18 WT were treated with cycloheximide (CHX). The cells were harvested after 12, 24, 36, 48, and 60 h, and the cell lysates were immunoblotted with antibody against Akt or actin. The graph represents the means ± S.E. of three independent experiments. (**E**) BHK-21 cells were transfected with Akt WT, K18 WT, and one of K8 WT or K8 G62C. The transfected cells were harvested at 12 h-intervals after CHX treatment. The data are represented as the means ± S.E. from three independent experiments. (**F**) The transfected BHK-21 cells were prepared as described in panel C. Akt activity was examined by immunoblotting of cell lysates with antibody against phosphorylated Akt T308. The relative Akt activity is shown in the graph as the means ± S.E. from three independent experiments. ‘n.s.’ indicates ‘not significant’.

K8/K18 mutations were identified in patients with liver diseases [28,29,30]. We examined whether the K8/K18 mutations have an effect on the K8/K18–Akt interaction. K8 G62C, R302C, and G434S mutants showed a statistically significant increase in K8/K18–Akt interaction compared with K8 WT (Figure 3A), while K18 mutants showed no significant change in their interaction (Figure 3B). Specifically, K8 G62C showed increased Akt binding with less variability as compared to K8 R302C or G434S mutants. A previous study demonstrated that the K8 G62C mutation interfered with the phosphorylation of K8 S74 and predisposed transgenic mice to liver toxins [31]. Unlike K8 G62C, K8 S74A (ser-74-ala, phosphorylation-blocked mutant) interacted with Akt as much as K8 WT (Figure 3C). In addition, K18 D238/397E (DEDE), which is resistant to caspase-mediated cleavage, was tested to measure the binding affinity of Akt to K8/K18, but its binding ability was not distinguishable to K18 WT (Figure 3B). Taken together, the K8/K18 binding to Akt was enhanced with some of the K8 mutants found in patients with liver diseases, specifically, K8 G62C.

### 2.4. K8/K18–Akt Interaction Tends to Stabilize Akt

We examined whether the interaction with K8/K18 affected Akt protein stability and Akt activity. Akt stability was compared in the BHK-21 cells, with or without expressing K8/K18, after cycloheximide (CHX) treatment for 60 h. K8/K18 expression in BHK-21 cells showed a trend to increase Akt stability, although it was not statistically significant (Figure 3D). Since K8 G62C mutation caused enhanced Akt binding (Figure 3A,C), we compared the Akt stability in BHK-21 cells expressing K8 WT/K18 WT with BHK-21 cells expressing K8 G62C/K18 WT. The K8 G62C expression tended to enhance the stability, although it was not statistically significant (Figure 3E). We then investigated whether the enhanced Akt stability led to the increased phosphorylation/activation of Akt. The BHK-21 cells were transfected with K8 WT or K8 G62C together with K18 WT and Akt WT, and then treated with OA. The cell lysates were prepared and immunoblotted against phosphorylated Akt T308. The result showed that the level of Akt T308 phosphorylation in K8 G62C transfected cells was similar to that in K8 WT transfected cells (Figure 3F). Taken together, K8/K18–Akt interaction might increase Akt protein stability but had no effect on Akt T308 phosphorylation in the in vitro cultured cell system.

### 2.5. K8/K18 Expression Enhances the Phosphorylation/Activation of Akt and Its Substrates in the In Vivo Mouse System

**Figure 4:**
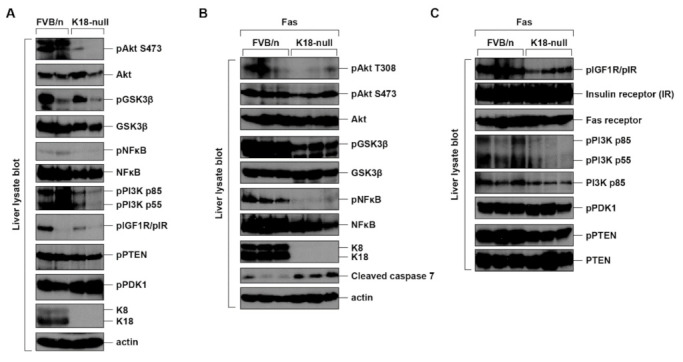
K8/K18 expression protects mouse liver from apoptosis by upregulation of Akt signaling pathway. (**A**) Livers from nontransgenic FVB/n and K18-null mice were harvested. The liver lysates were immunoblotted with the indicated antibodies. Sex- and age-matched mice were used, and each lane represents the result of one individual mouse liver. (**B**,**C**) Sex- and age-matched nontransgenic FVB/n or K18-null mice were treated with Fas (0.15 µg/g body weight) for 4 h to induce liver damage. The liver lysates were immunoblotted with the indicated antibodies. Each lane represents the result of one individual mouse liver.

A previous study demonstrated that K18-null mice were highly susceptible to Fas-mediated liver damage, as compared to the nontransgenic mice [6]. K18-null mice did not express endogenous mouse K18, but did express mouse K8, which was rapidly degraded, as the lack of K18 did not result in the formation of a stable K8/K18 heterodimer. Hence, the K8/K18 proteins were detected in the livers of nontransgenic FVB/n (Friend leukemia virus B/n) mice but not in those of K18-null mice (Figure 4A). Although K8/K18-mediated liver protection was described in the studies using K8- or K18-null mice, it is not fully reported whether the Akt signaling pathway is involved in the protection of the liver under Fas-induced damage. We compared the effect of K8/K18 expression on the phosphorylation/activation of Akt in the control FVB/n and K18-null mice after Fas treatment. The total liver lysates were prepared and immunoblotted with indicated antibodies (Figure 4). Under basal conditions, the level of Akt S473 phosphorylation in control livers was higher than that in K18-null livers (Figure 4A). The variability of an individual mouse in the same strain of mouse was observed (lanes 1 and 2 in Figure 4A). However, the higher levels of cleaved caspase 7 were consistently observed in K18-null livers compared with control livers after Fas treatment, indicating that K18 livers had more severe apoptosis (Figure 4B).

Next, we compared the phosphorylation/activation of signaling molecules in Fas-treated livers. Among the tested signaling molecules, the higher phosphorylation levels of Akt T308 and Akt substrates, such as GSK3β and NFκB, were detected in the control livers compared with the K18-null livers (Figure 4B), while the phosphorylation levels of other tested signaling proteins showed a limited change in both livers (Figure 4C). Notably, it seemed that the amount of NFκB itself was higher for FVB/n compared to K18-null (Figure 4B). We compared the ratio between pNFκB and NFκB from three mice per each strain. The ratio of pNFκB to NFκB in control (K18 expressing) FVB/n livers was 0.52 ± 0.1, while the ratio in K18-null livers was 0.15 ± 0.09. Therefore, the phosphorylation of NFκB was enhanced in the K18-expressing conditions compared to the K18-absent conditions. In addition, a previous study showed the K8/K18 binding with NFκB [32]. Although the effects of the interaction have not been revealed yet, it is likely, at least in part, that K8/K18-NFκB interaction tends to stabilize NFκB protein. In summary, the interaction between Akt and K8/K18 is regulated by keratin phosphorylation, specifically, K18 phosphorylation, but their binding does not have an effect on the alteration of Akt activity in the cultured cell system. However, K8/K18 expression in the mouse system is associated with the upregulation of the Akt signaling pathway, which might be involved in the protection of the liver from damage.

## 3. Discussion

### 3.1. Akt-Mediated Resistance to Fas-Induced Apoptosis

Herein, we verified that Akt had a higher affinity with the K18 phosphorylation-deficient mutant and K8 mutants, specifically, K8 G62C, from liver patients, but the increased interaction did not change Akt activity. In addition, we found that K8/K18 are potential substrates of Akt, but we did not observe that Akt phosphorylated on K8 T26 or reported phosphorylation serine residues under testing conditions. However, Akt activity is upregulated in the cells with K8/K18 expression compared with the activity in the cells without K8/K18 expression. It seems that K8/K18 expression itself has an effect on the regulation of Akt.

K18-null mice were highly susceptible to liver-specific stresses, such as Fas and microcystin [6]. It was suspected that the high susceptibility of K18-null mice might be caused by downregulated PI3K/Akt activity. Although a direct correlation was not reported, previous studies have demonstrated that the Akt pathway is involved in survival regulation under Fas treatment conditions [33,34] and that the Akt pathway blocks apoptosis through NF-κB in mouse primary hepatocytes [35]. Our study showed the significance of K8/K18 expression in the regulation of the Akt pathway. During liver damage, the phosphorylation/activation of Akt and its substrates, such as GSK3β and NFκB, was downregulated in K18-null mice, which led to increased apoptosis in K18-null livers (Figure 4). Our study may represent a possible mechanism of liver protection that describes the role of K8/K18 in the Akt signaling pathway.

### 3.2. A Role of K8/K18 in Tumor Progression

We considered Akt to prevent tissue damage in this study. However, the aberrant activation of the PI3K/Akt pathway frequently occurs in different types of cancers [36,37]. Since the PI3K/Akt pathway controls cell survival, proliferation, and growth, the dysregulated activation of the pathway can accelerate cancer development [37,38]. The overexpression of PI3K and Akt was observed in ovarian, breast, and pancreatic cancers [38].

K8/K18 are known as a biomarker for several tumors, since keratins are detected in the circulating tumor cells (CTCs) in blood and the protein level of K8/K18 is increased in tumor patients [39,40]. Since the roles of K8/K18 in tumor development have not been elucidated precisely, the increase in K8/K18 has been considered as a tumor marker, and thus, K8/K18 has been used for diagnosis. However, a previous study reported that the knockdown of K8 reduced the tumorigenicity of A431 cells [41]. Given that K8 dimerizes with K18 and the stable K8/K18 complex is formed, the amount of K18 is decreased under K8 knockdown conditions as the expressed K18 is unstable and degraded. The study of A431 cells indicates that K8/K18 might be able to induce tumor development. Moreover, our study showed that K8/K18–Akt interaction tended to enhance Akt stability and K8/K18 expression increased Akt activity in the mouse system. Although keratin-mediated tumorigenesis and its related mechanisms were not fully investigated, it seemed that the increased K8 or K18 expression in tumors might be correlated with aberrant Akt activation.

In the current study, it was revealed that Akt-K8/K18 interaction was affected by keratin phosphorylation, specifically, K18 phosphorylation, and K8/K18 expression increased the phosphorylation of Akt and its substrates, such as GSK3β and NFκB. Since the Akt signaling pathway is highly related to tumorigenesis as well as cell survival, the tight regulation of the Akt signaling pathway is critical for the treatment of tumors. Our study demonstrated the possibility of K8/K18 involvement in the regulation of the Akt signaling pathway. Hence, elucidating the roles of K8/K18 might be significant for finding new targets for Akt regulation.

## 4. Material and Methods

### 4.1. List of Reagents and Antibodies

The following reagents were used in this study: okadaic acid (OA) (ALS-350-003; Enzo Life Sciences, Farmingdale, NY, USA), MS-275 (ALX-270-378; Enzo Life Sciences, NY, USA), trichostatin A (TSA) (T8552; Millipore Sigma, Burlington, MA, USA), nicotinamide (N3376; Millipore Sigma), hemin (51280; Millipore Sigma), carbon monoxide-releasing molecule (CORM) (288144; Millipore Sigma), and cycloheximide (CHX) (C1988; Millipore Sigma).

The following antibodies were used in this study (Supplementary Table S1): L2A1 mouse monoclonal antibody [42]; anti-K8/K18 rabbit polyclonal antibody 8592 [43]; anti-K8 R341 antibody 2078 [44]; phospho-Akt T308, phospho-Akt S473, Akt, phospho-IGF1R/IR, insulin receptor β, phospho-PI3K, PI3K, phospho-PTEN, PTEN, phospho-PDK1, phospho-NFκB, NFκB, phospho-GSK3β, GSK3β, cleaved caspase 7, phosphorylated Akt substrate (PAS) (Cell Signaling Technology, Danvers, MA, USA), and actin (Invitrogen, Waltham, CA, USA).

### 4.2. Cell Transfection and Preparation of Cell/Tissue Lysates

Human colorectal adenocarcinoma cell line (HT29) and baby hamster kidney fibroblast cell line (BHK-21) were maintained in growth media (RPMI and DMEM, respectively) containing FBS and penicillin/streptomycin. HT29 cells were treated with okadaic acid (OA, 1 µg/mL for 2 h), TSA/MS-275/nicotinamide (2 µM/5 µM/20 mM for 24 h), or CORM/hemin (100 µM/10 µM for 10 h) to increase the levels of phosphorylation, acetylation, and methylation, respectively.

BHK-21 cells were seeded with 2 × 10^6^ cells in 100 mm-dishes a day before the transfection and transfected by jetPRIME (Polyplus-transfection, New York, NY, USA) according to the protocol provided by the company. The cells were treated with OA (0.5 µg/mL) for 2 h. The collected cells were lysed in SDS-containing sample buffer to prepare the total lysates. In the case of experiments with transgenic mice, liver tissue was homogenized by a tissue grinder, and the tissue homogenate was mixed with condensed sample solution to prepare the total liver lysates.

### 4.3. Co-Immunoprecipitation and Immunoblot Analysis

Cells were lysed with 1% IGEPAL CA-630 (I8896; Sigma-Aldrich, St. Louis, MO, USA) or 0.5% empigen (30325; Sigma-Aldrich, MO, USA)/PBS containing 10 mM EDTA, 5 mM sodium pyrophosphate, and 50 mM sodium fluoride with a protein inhibitor cocktail for 5 h, and the lysates were separated by centrifugation at 14,000 rpm for 20 min [45]. Co-immunoprecipitation was performed by incubating L2A1 antibody-attached G protein beads or the indicated antibodies with the supernatants.

The immunoprecipitates or the total lysates were separated on acrylamide gels by SDS-PAGE and then transferred to the PVDF membrane. The membrane was blocked by 5% milk in tween20/PBS and then incubated with a primary antibody (1:500~1:1000 dilution) and a secondary antibody (1:2000 dilution) in order in the shaking incubator at room temperature. The proteins were visualized by the enhanced chemiluminescence system.

### 4.4. Protein Stability Analysis

BHK-21 cells, which were transfected with K8/K18 WT or mutants, were split into 24-well dishes. One day later, the cells were treated with CHX (100 µg/mL), and then they were harvested at every 12 or 24 h. The cell lysates were immunoblotted with an antibody against K8 or Akt.

### 4.5. Animals and TISSUE Collections

Keratin 18 was knocked out in FVB/n background mice. Nontransgenic FVB/n mice and K18 null mice were fed with laboratory food and water then starved for 24 h before intraperitoneal injection of Fas antibody (0.15 µg/g body weight). After 4 h, mice were euthanized with CO_2_, and livers were isolated. The animal experiments followed the Korean Food and Drug Administration (KFDA) guidance, and the protocols for the experiments were approved by the Institutional Animal Care and Use Committee (IACUC) of Yonsei University.

### 4.6. Site-Directed Mutagenesis

K8 or K18 wild-type gene was subcloned into downstream of the hCMV promoter in pMRB101 mammalian expression vector, and then the plasmids were used for generation of K8 or K18 mutant. Plasmids containing K8 or K18 mutations were generated by the PCR-based mutagenesis method. PCR was performed with designed primers using Pfu high-fidelity polymerase (Agilent Technologies, Santa Clara, CA, USA).

### 4.7. Statistical Analysis

Densitometry was performed with ImageJ bundled with 64-bit Java 1.8.0_172 (https://imagej.nih.gov/ij/, accessed on 11 May 2021), and the data were statistically analyzed using Student’s *t*-test. A *p*-value less than 0.05 was considered statistically significant.

## Supplementary Materials

The following are available online at https://www.mdpi.com/article/10.3390/ijms22179227/s1.

## Abbreviations

cycloheximide, CHX; IκB kinase, IKK; keratin, K; okadaic acid, OA; phosphorylated Akt substrate, PAS; phosphoinositide 3-kinase, PI3K; phosphatidylinositol 4,5-bisphosphate, PIP_2_; phosphatidylinositol 3,4,5-triphosphate, PIP_3_; protein kinase A, PKA; protein kinase B, PKB; protein kinase C, PKC; wild type, WT.
