# Supplementary material for: Keratin 8/18 Regulate the Akt Signaling Pathway
Source: Int J Mol Sci. 2021 Aug 26;22(17):9227. doi: 10.3390/ijms22179227 (PMC8430995; doi:10.3390/ijms22179227)
Supplement: Supplementary file 1 [file ijms-22-09227-s001.zip › ijms-1325821-supplementary.pdf]

Supplementary Figure S1

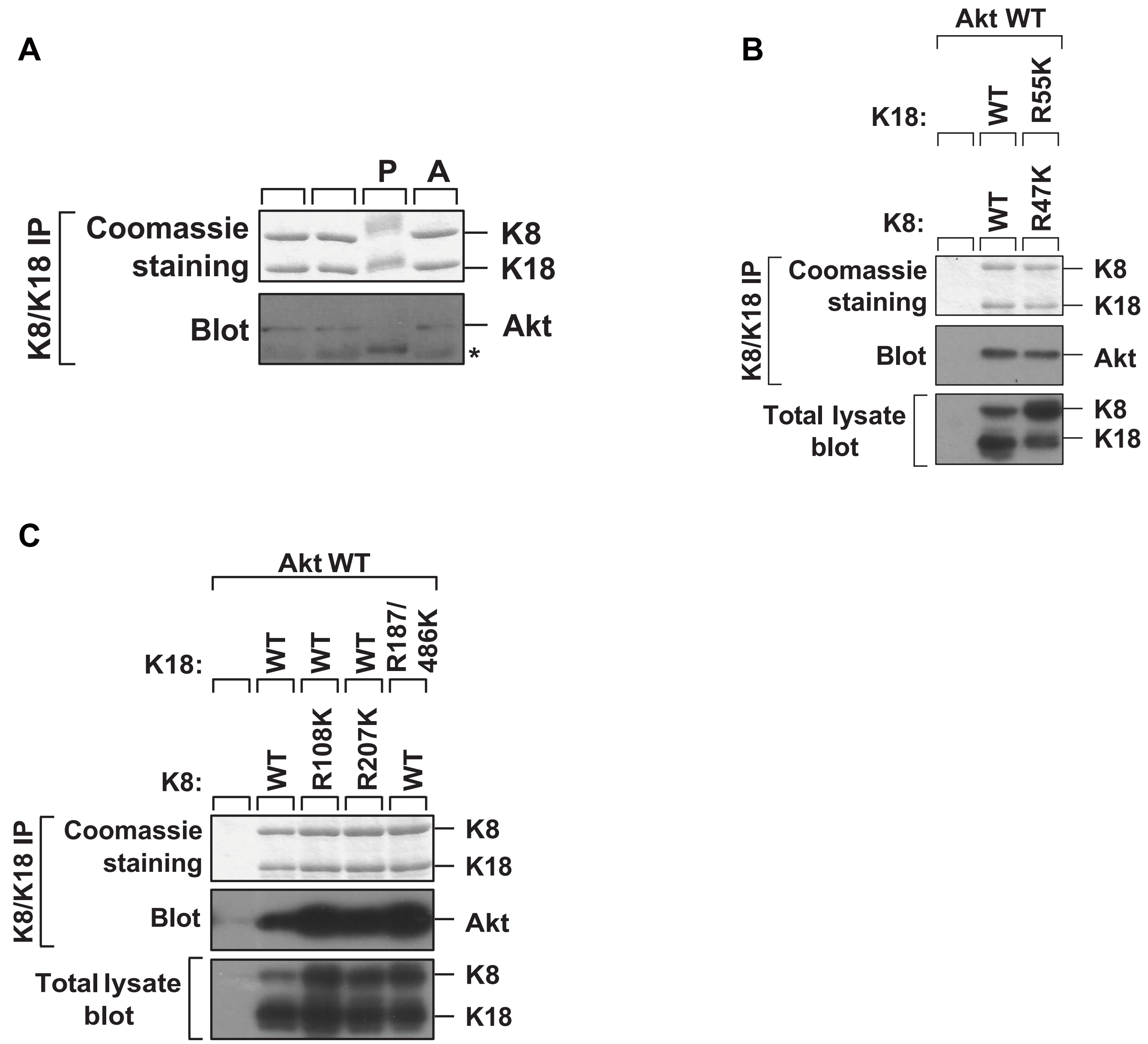

Supplementary Figure S1. K8/K18–Akt interaction is altered depending on phosphorylation, but not methylation or acetylation. (A) HT29 cells were treated with methylation enhancers (CORM/hemin; 100 and 10 uM) for 10 hours, deacetylase inhibitors (TSA/MS-275/nicotinamide; 2, 5, and 20 mM) for 24 hours, and a phosphatase inhibitor (OA; 1 ug/ml) for 2 hours. The cells were collected and immunoprecipitated against K8/K18, followed by Coomassie blue staining or immunoblotting against Akt. (B) BHK-21 cells were transfected with K8/K18 WT or methylation site mutants (K8 R47K/K18 R55K). The transfected cells were lysed, and the cell lysates were immunoprecipitated with antibody against K8/K18. The K8/K18 immunoprecipitates were blotted with antibody against Akt. (C) BHK-21 cells were transfected with K8/K18 WT or acetylation site mutants (K8 R108K, R207K, or K18 R187/486K). The transfected cells were analyzed as described in panel B.

Supplementary Table S1. List of antibodies.

Target protein	Manufacturer	Cat #
Actin	Thermo Fisher Scientific	MA5-11869
Akt	Cell Signaling Technology	9272
Akt	Cell Signaling Technology	2966
Phospho-Akt T308	Cell Signaling Technology	9275
Phospho-Akt S473	Cell Signaling Technology	9271
Insulin receptor β	Cell Signaling Technology	3025
Phospho-IGF1 receptor β / Insulin receptor β	Cell Signaling Technology	3024
PI3K	Cell Signaling Technology	4292
Phospho-PI3K	Cell Signaling Technology	4228
PTEN	Cell Signaling Technology	9559
Phospho-PTEN	Cell Signaling Technology	9551
Phospho-PDK1	Cell Signaling Technology	3061
NFκB	Cell Signaling Technology	4764
Phospho-NFκB	Cell Signaling Technology	3031
GSK3β	Cell Signaling Technology	9315
Phospho-GSK3β	Cell Signaling Technology	9336
Cleaved caspase 7	Cell Signaling Technology	9491
Phosphorylated Akt substrate (RXRXXpS/T)	Cell Signaling Technology	10001
Phosphorylated Akt substrate (RXXpS/T)	Cell Signaling Technology	9614
